# Genome Sequence of Kosakonia radicincitans Strain YD4, a Plant Growth-Promoting Rhizobacterium Isolated from Yerba Mate (Ilex paraguariensis St. Hill.)

**DOI:** 10.1128/genomeA.00239-15

**Published:** 2015-04-02

**Authors:** Veronica M. Bergottini, Sevasti Filippidou, Thomas Junier, Shannon Johnson, Patrick S. Chain, Monica B. Otegui, Pedro D. Zapata, Pilar Junier

**Affiliations:** a Laboratory of Microbiology, Neuchâtel, Switzerland; b Vital-IT Group, Swiss Institute of Bioinformatics, Lausanne, Switzerland; c Los Alamos National Laboratory, Los Alamos, New Mexico, USA; d Instituto de Biotecnología, Misiones, Argentina

## Abstract

Kosakonia radicincitans strain YD4 is a rhizospheric isolate from yerba mate (Ilex paraguariensis St. Hill.) with plant growth-promoting effects on this crop. Genes involved in different plant growth-promoting activities are present in this genome, suggesting its potential as a bioinoculant for yerba mate.

## GENOME ANNOUNCEMENT

Yerba mate is an important southern South American crop, used to produce an energizing beverage, widely consumed in this region as an alternative to coffee. Due to mate tea’s high antioxidant content and nutritional benefits for human health, its popularity is rapidly increasing, with expansion to new markets, including Europe, Asia, and the United States (1). Argentina is the leading producer of yerba mate with plantations mainly clustered around the Northeast of the country. Currently, producers are struggling with soil degradation due to inadequate agricultural practices. Bioinoculation of yerba mate with native bacterial isolates was recently demonstrated as a sustainable agricultural practice to improve plant growth (V. Bergottini, M. Otegui, D. Sosa, P. Zapata, M. Mulot, M. Rebord, J. Zopfi, F. Wiss, B. Benrey, and P. Junier, submitted for publication).

The sequenced bacterium was isolated from the rhizosphere of yerba mate in a soil-degraded plantation in Argentina. The strain YD4 was selected on the basis of its plant growth-promoting effect on yerba mate seedlings growing in a nursery, reflecting its potential as a bioinoculant (Bergottini et al., submitted). The 16S rRNA, *atp*D and *rpo*B gene sequences of YD4 showed the highest homology with the plant growth-promoting endophyte Kosakonia radicincitans DMS16656^T^ (2). The sequencing and annotation of the genome aimed at a broader understanding of the plant-growth promotion mechanisms and the safety verification for using this strain as a bioinoculant.

Genomic DNA was extracted from an overnight culture using the Genomic-tip 20/G kit (Qiagen GmbH, Germany). Sequencing was performed with the PacBio RS II system based on single molecule, real-time (SMRT) technology (Pacific Biosciences, California). The draft genome of K. radicincitans YD4 presents a unique contig of 5,226,863 bases, and a G+C content of 54.3%. Genome annotation was performed using an Ergatis-based (3) workflow with minor manual curation and visualized with Artemis Genome Browser and Annotation Tool (4). A total of 4,836 coding sequences (CDSs), 82 tRNAs, and 7 rRNAs were predicted.

The plant growth-promoting abilities of the strain YD4 experimentally observed were consistent with the presence of genes for nitrogen fixation (*nifQBALFMZWVSUXNEYTKDHJ*), for the mineralization of organic phosphates (*phoC*), siderophore production (*entABCDEF*) and uptake (*fepABCDG* and *fhuFCDBE*), an indolpyruvate decarboxylase (*ipdC*) involved in the synthesis of the auxin indol acetic acid (IAA), and an auxin efflux carrier. Most of these genes were also described in the K. radicincitans DMS16656^T^ genome (2). Similar to the endophytic nitrogen-fixing strain Enterobacter sp. SP1 (5), K. radicincitans YD4 presents genes for the type III secretion system, suggesting that a broader analysis of these genes will be necessary in order to infer a potential mutualistic or parasitic lifestyle in this strain.

### Nucleotide sequence accession numbers.

This whole-genome shotgun project has been deposited at DDBJ/EMBL/GenBank under the accession no. JSFC00000000. The version described in this paper is version JSFC01000000.
